# Repurposing the Bis-Biguanide Alexidine in Combination with Tyrosine Kinase Inhibitors to Eliminate Leukemic Stem/Progenitor Cells in Chronic Myeloid Leukemia

**DOI:** 10.3390/cancers15030995

**Published:** 2023-02-03

**Authors:** Fabien Muselli, Lucas Mourgues, Nathalie Rochet, Marielle Nebout, Agnès Guerci, Els Verhoeyen, Adrien Krug, Laurence Legros, Jean-François Peyron, Didier Mary

**Affiliations:** 1 Institut National de la Santé et de la Recherche Médicale (Inserm) U1065, Centre Méditerranéen de Médecine Moléculaire, Université Côte d’Azur, Team 4, CEDEX 03, 06204 Nice, France; 2 Institut de Biologie Valrose, Université Côte d’Azur, CNRS UMR 7277, Inserm U1091, CEDEX 02, 06107 Nice, France; 3 Hematology Department, University Hospital, 54000 Nancy, France; 4 Department of Hematology, Paul Brousse Hospital, 94000 Créteil, France

**Keywords:** chronic myeloid leukemia, leukemic stem/progenitor cells, oxidative metabolism, apoptosis, BMI1, alexidine

## Abstract

**Simple Summary:**

Over the past two decades, three generations of Tyrosine Kinase Inhibitors (TKIs) have been developed to target the BCR::ABL1 oncoprotein to successfully treat chronic myeloid leukemia (CML). However, to maintain a definitive remission state, the daily administration of TKIs cannot be stopped, generating many unwanted side effects in the long term. Moreover, half of the patients will relapse after cessation of TKIs, highlighting the persistence of undetectable TKI-insensitive leukemic stem cells (LSCs). In recent years, new pharmacological approaches have been proposed to improve the sensitivity of LSCs to TKIs. We demonstrate here that bis-biguanides combined with TKIs can efficiently eliminate CML LSCs.

**Abstract:**

Background & aims: In CML, Leukemic Stem Cells (LSCs) that are insensitive to Tyrosine Kinase Inhibitors are responsible for leukemia maintenance and relapses upon TKI treatment arrest. We previously showed that downregulation of the BMI1 polycomb protein that is crucial for stem/progenitor cells self-renewal induced a CCNG2/dependent proliferation arrest leading to elimination of Chronic Myeloid Leukemia (CML) cells. Unfortunately, as of today, pharmacological inhibition of BMI1 has not made its way to the clinic. Methods: We used the Connectivity Map bioinformatic database to identify pharmacological molecules that could mimick BMI1 silencing, to induce CML cell death. We selected the bis-biguanide Alexidin (ALX) that produced a transcriptomic profile positively correlating with the one obtained after BMI silencing in K562 CML cells. We then evaluated the efficiency of ALX in combination with TKI on CML cells. Results: Here we report that cell growth and clonogenic activity of K562 and LAMA-84 CML cell lines were strongly inhibited by ALX. ALX didn’t modify BCR::ABL1 phosphorylation and didn’t affect BMI1 expression but was able to increase CCNG2 expression leading to autophagic processes that preceed cell death. Besides, ALX could enhance the apoptotic response induced by any Tyrosine Kinase Inhibitors (TKI) of the three generations. We also noted a strong synergism between ALX and TKIs to increase expression of caspase-9 and caspase-3 and induce PARP cleavage, Bad expression and significantly decreased Bcl-xL family member expression. We also observed that the blockage of the mitochondrial respiratory chain by ALX can be associated with inhibition of glycolysis by 2-DG to achieve an enhanced inhibition of K562 proliferation and clonogenicity. ALX specifically affected the differentiation of *BCR::ABL1*-transduced healthy CD34^+^ cells but not of mock-infected healthy CD34^+^ control cells. Importantly, ALX strongly synergized with TKIs to inhibit clonogenicity of primary CML CD34^+^ cells from diagnosed patients. Long Term Culture of Initiating Cell (LTC-IC) and dilution of the fluorescent marker CFSE allowed us to observe that ALX and Imatinib (IM) partially reduced the number of LSCs by themselves but that the ALX/IM combination drastically reduced this cell compartment. Using an in vivo model of NSG mice intravenously injected with K562-Luciferase transduced CML cells, we showed that ALX combined with IM improved mice survival. Conclusions: Collectively, our results validate the use of ALX bis-biguanide to potentiate the action of conventional TKI treatment as a potential new therapeutic solution to eradicate CML LSCs

## 1. Introduction

The concept of targeted therapy emerged 20 years ago with the introduction of the Tyrosine Kinase Inhibitor (TKI) imatinib mesylate (IM), which targets the BCR:BCR:ABL1 oncoprotein [1] as a treatment for chronic myeloid leukemia (CML). Two new generations of TKIs were developed to replace imatinib when it was either poorly tolerated or ineffective. These new TKIs can be distinguished as mono [2] or dual TKIs, the latter targeting other kinases in addition to BCR::ABL1, such as SRC-family kinases [3,4] and Aurora kinases [5], which may explain their differential effects in patients. However, none of them seems to be sufficiently effective to treat the disease in the long term, the main reason being the lack of sensitivity of leukemic stem cells (LSCs) to these TKIs, as these cells do not rely on BCR::ABL1 [6,7]. Indeed, LSCs were demonstrated to be only slightly or not at all sensitive to TKIs, whether imatinib [8], dasatinib [9], nilotinib [10], or bosutinib [11]. At present, many studies are focusing on the targeting of molecular pathways that are essential to LSCs’ maintenance/persistence in conjunction with conventional TKI treatment. For instance, targeting of the N-cadherin/Wnt-β-catenin pathway [12], the hedgehog pathway [13], the protein kinase C delta [14], HIF [15,16], the mitochondrial function [17], or STAT5 [18,19]—all of which are involved in the maintenance of LSCs—has proven some efficacy, some of them having led to clinical trials.

We previously reported that downregulation of BMI1, which is well known to support self-renewal and maintenance of LSCs in CML, reduced proliferation and clonogenicity and induced autophagy [20]. We demonstrated that expression of the tumor-suppressor gene *CCNG2 (CyclinG2)* after BMI1 silencing was important for the cell death response of LCSs [20]. Here, we compared the transcriptome of BMI1-silenced K562 cells with those contained in the Connectivity Map database of perturbagen-induced transcriptomic profiles on the HL-60 leukemic cell line. This analysis identified several molecules, including the bis-biguanide alexidine (ALX). It has been known for several years that molecules of the biguanide family, such as metformin and phenformin, are of potential interest in oncology [21]. ALX, which was first used as an oral disinfectant, was also successfully tested for neck and head cancers [22] and oral cell tumors [23]. More recently, ALX was demonstrated to induce apoptosis in human pancreatic adenocarcinoma cell lines [24]. The screening of 1170 FDA-approved drugs on malignant pleural mesothelioma cell lines has also highlighted ALX as a potential candidate to affect these cancerous cells [25].

We thus explored the effects of ALX on both CML lines and primary CML cells, demonstrating that the use of ALX together with a TKI can lead to the efficient elimination of LSCs.

## 2. Materials and Methods

### 2.1. Reagent and Antibodies

RPMI 1640 medium, penicillin, streptomycin, sodium pyruvate, and fetal calf serum were purchased from Gibco (Invitrogen Life Technologies, Waltham, MA, USA). Sodium fluoride, sodium orthovanadate, aprotinin, leupeptin, phenylmethylsulfonyl fluoride, phorbol 12-myristate 13-acetate, SB202190, Triton X-100, Dasatinib, Bosutinib and alexidine were purchased from Sigma. Imatinib mesylate was purchased from Enzo Life Sciences, nilotinib from Selleck Chemicals, and ponatinib from Clinisciences. The antibodies used in these studies are listed in Table 1.

### 2.2. Cell Lines

The human CML cell lines LAMA-84 and K562 were obtained from ATCC. They were cultured in RPMI 1640/5% fetal calf serum (FCS)/1 mM sodium pyruvate/50 U per mL penicillin/50 μg per mL streptomycin and regularly tested for mycoplasma contamination (MycoAlert Mycoplasma Detection Kit-Lonza #LT07-418).

### 2.3. Proliferation Assay

Cells (4 × 10^5^ cells seeded per well) were grown and treated on 12-well plates. The IncuCyte Zoom Imaging system (Essen Bioscience, Ann Arbor, MI, USA) was used for confluence counting by capturing images of the culture dish every two hours for 24 h. The optical density of the cells was measured in 9 squares per well (in triplicate), and the IncuCyte^®^ Zoom v2018A software (Sartorius Stedim Biotech GmbH, Goettingen, Germany) was used to analyze the images and determine the confluence index as the percentage of the dish that was covered by the cells.

### 2.4. Viability Assay and Synergy Calculation Scores

Cell viability was determined by DAPI staining prior to flow cytometry analysis in 96-well plates using MACSQuant or MACSQuant VYB flow cytometers (Miltenyi Biotec, Bergisch Gladbach, Germany). Absolute numbers of DAPI-negative cells were determined, and the treatment conditions were normalized to controls.

Synergy scores were calculated using the SynergyFinder software (https://synergyfinder.fimm.fi) accessed on 23 may 2022. Briefly, the synergy scores for the drug combinations were averaged over all of the measurements of the dose combinations. To quantify the degree of synergy, the highest single agent (HSA) model was employed. Dose–response curves were fitted with LL4 (four-parameter logistic regression), and a readout viability baseline correction was applied.

### 2.5. Analysis of Apoptosis

Annexin V–DAPI staining was used to detect apoptosis. Briefly, cells were washed with PBS/2% FCS, and then stained in FITC–annexin-V-containing buffer (Miltenyi, 130-097-928, Bergisch Gladbach, Germany) and DAPI (Sigma, D9542-5MG, St. Louis, MO, USA) according to the manufacturer’s protocol. The cells were washed, resuspended in PBS/2% FCS, and analyzed by flow cytometry (MACSQuant 10, Miltenyi, 130-097-928, Bergisch Gladbach, Germany). FlowJo^®^ software (v10) (BD Biosciences Le Pont-de-Claix, France) was used for analyzing these data.

### 2.6. May–Grünwald–Giemsa (MGG) Staining

Cells were attached onto a microscope slide (10 min at 800 g) in a Cytospin 4 apparatus (Shandon Thermo Electron Corp, Waltham, MA, USA). The slides were then air-dried and stained with MGG reagents from Sigma (St. Louis, MO, USA).

### 2.7. Confocal Microscopy

K562 cells were stably transfected with a plasmid encoding LC3-RFP (red fluorescent protein) [26]. Cells were attached on microscope slides (10 min at 800 g) and then air-dried, fixed with 4% paraformaldehyde, and permeabilized with 0.1% Triton X-100. The cells were then incubated with specific conjugated antibodies and mounted on coverslips, before analyzing them with a confocal microscope (Zeiss LSM 510 Meta, Oberkochen, Germany).

### 2.8. Isolation of Primary Cells 

Bone marrow biopsies were collected from newly diagnosed CML patients as part of an institutionally approved cellular sample collection protocol (Centre Hospitalier, Nice, France). CD34^+^ cell samples from bone marrow or cord blood were collected from healthy donors after informed consent. Mononuclear cells were isolated by density centrifugation (Ficoll-Paque Plus, StemCell Technologies, Vancouver, BC, Canada) and washed with PBS/5% fetal calf serum (FCS) followed by incubation with CD34 microbeads (CD34 MicroBead Kit, Miltenyi Biotec) before sorting. Purity was determined by incubation with anti-human CD34 Ab (Miltenyi, 130-113-741) and analysis by flow cytometry (purity should be more than 96%).

### 2.9. Colony-Forming Cell (CFC) Assay

Human cell lines were seeded in methyl cellulose medium (STEM MACSQ HCF-CFU Basic, Miltenyi Biotec) on 12-well plates (3 × 10^3^ cells/well) in the presence of drugs at indicated concentrations. After 7 days, colonies were labeled with the tetrazolium salt MTT and quantified with ImageJ software v 1.53a (National Institutes of Health, USA).

Primary CD34^+^ CML cells were seeded in serum-free medium (HPC Expansion medium from PromoCell, Heidelberg, Germany) in the presence of drugs at indicated concentrations. After 48 h, cells (0.9 × 10^3^) from each condition were transferred in 1 mL of alpha-MEM based methylcellulose medium (GF H4434, StemCell Technologies) to 35 mm tissue culture dishes. CFCs were scored after 14 days of incubation at 37 °C and 5% CO_2_.

### 2.10. Cellular Division Tracking

CD34+ cells from diagnosed CML patients were stained with 2 μM 5-(and 6-) carboxyfluorescein diacetate succinimidyl diester (CFSE, Invitrogen) for 15 min. After washing, the cells were seeded at 5 × 10^5^/mL in StemSpan SFEM medium (StemCell Technologies) without cytokines and with or without alexidine, imatinib, or their combination, as indicated. Cells were harvested at 7 days and labeled with anti-CD45 and anti-CD34 before analysis by flow cytometry.

### 2.11. Long-Term Culture-Initiating Cell (LTC-IC) with Limiting Dilution Assays (LDAs)

LTC-IC with LDAs were performed in StemSpan SFEM medium (StemCell Technologies) on irradiated stromal M2.10B4 monolayers at several dilutions of CD34^+^ cells (300, 150, 75, or 37 cells per well for CML CD34^+^ cells, and 200, 100, 50, or 25 cells per well for CD34^+^ from healthy donors) in 96-well plates, with 16 replicate wells per concentration. The cells were kept for a minimum of 5 weeks, with half of the medium changed once per week. The remaining cells were assessed by CFC, and frequencies were calculated using L-Calc^TM^ software v1.1 (StemCell Technologies).

### 2.12. Plasmids and Transduction

Generation of the lentiviral vector encoding BCR::ABL1: The PCR-amplified BCR::ABL1 product was subcloned into a pCR(R)-XL TOPOR plasmid (Invitrogen Life Technologies) before being inserted into the SIV-GAE-SFFViresGFP lentiviral vector [14]. The SIV GAE-SFFViresGFP vector was used as a control. The SIV vectors were produced as previously described [14]. For transduction of CD34+ cells, cells were seeded (5 × 10^4^/mL) in StemSpan medium (StemCell Technologies) in a 96-well plate coated with RetroNectinR (Takara Shuzo Co., Kyoto, Japan), to which protamine sulfate (4 µg/mL), SCF (100 ng/mL), Flt-3-L (100 ng/mL), IL-3 (20 ng/mL), and IL-6 (20 ng/mL) were added. After 16 h of stimulation, lentiviral vectors were added to the CD34+ cells for 12 h. The cells were washed twice and placed in fresh medium. At day 3 post-transduction, GFP+ cells were detected using flow cytometry.

For generating transduced K562 cells, self-inactivating lentiviral vectors were generated by transient transfection of 293 T cells and titered as described previously [27]. Briefly, using the calcium phosphate method, the envelope plasmid VSV-G (3 μg) was co-transfected with 8.6 μg of Gag-Pol packaging plasmid (psPAX2, Addgene, #12260) and 8.6 μg of a lentiviral vector coding for a fusion protein between luciferase and GFP (HIV-SFFV-LUCI-GFP). Eighteen hours after transfection, the medium was replaced with Opti-MEM supplemented with 1% HEPES (Invitrogen). Viral supernatants were harvested 48 h after transfection and filtered with a 0.45 μm filter. The vectors were concentrated at low speed by overnight centrifugation of the viral supernatants at 3000× *g* and 4 °C. Viral particles were titered in K562 cells by serial dilutions. K562 cells were transduced with viruses at a multiplicity of infection (MOI) of 1. Cells were kept for up to 48 h before medium refreshment and cell expansion for in vivo injection.

### 2.13. Western Blot

For protein extraction, the following lysis buffer was used: 50 mM HEPES pH 7.4, 150 mM NaCl, 20 mM EDTA, 100 µM NaF, 10 mM Na3VO4, 1% Nonidet P-40 and protease inhibitors. Proteins were separated on a 10% SDS-PAGE gel. After Western blotting and antibody (see Table 1) incubation, ECL detection (Amersham Pharmacia, Amersham, UK) and light signals were recorded as previously described [20]. The uncropped Western blots are in a supplementary file.

### 2.14. Oxygen Consumption Rate Measurements

Real-time oxygen consumption rate (OCR) measurements were performed using a Seahorse XFe96 extracellular flux analyzer (Agilent Technologies, Santa Clara, CA, USA). Briefly, K562 cells or human CD34+ cells were seeded in 96-well plates coated with Cell-Tak at a density of 50,000 cells or 100,000 cells per well, respectively, in 180 μL of XF base medium DMEM (Agilent Technologies) completed with 20 mM D-glucose, 1 mM sodium pyruvate, and 2 mM L-glutamine at pH 7.4. For cell adhesion, the culture plates were centrifuged at 200× *g* and incubated at 37 °C for 20 min. Real-time OCR measurements were processed after serial injections of mitochondrial inhibitors (1 μM oligomycin, 0.5 μM FCCP, and 1 μM rotenone combined with 1 μM of antimycin A).

### 2.15. Statistical Analysis

For statistical analyses, Prism 6 was used (GraphPad Software, Version 6.0, San Diego, CA, USA). When comparing 2 independent experimental conditions, *t*-tests were performed. For more than 2 independent data groups, two-way ANOVA with Tukey’s post hoc test was employed. Two-tailed tests were used throughout; asterisks indicate the designated significance (* *p* < 0.05; ** *p* < 0.01; *** *p* < 0.001; **** *p* < 0.0001).

### 2.16. In Vivo Experiments:

NSG transgenic mice were bred and housed in the local animal facility (C3M, Nice France).

First, 5 × 10^5^ K562 cells transduced with the HIV-SFFV-LUCI-GFP vector were injected IV into 6-week-old NSG mice. Treatments were initiated one day after cell injection for each group (7 mice/group). The mice were weighed every 2 days, and drugs were injected intraperitoneally every day for 10 days. Leukemogenesis was evaluated by bioluminescence analysis (Optima Biospace, Biospace Lab, Nesles-la-Vallée, France) according to the manufacturer’s instructions. Acquisition and analysis were performed using Biospace Lab Photo acquisition 2.8 and M3 Vision software, respectively. Image acquisitions were matched to photonic signals integrated for 5 min. BLI signals were quantified and expressed as photon/cm^2^/steradian (ph/cm^2^/Sr).

Mice were injected intraperitoneally with 3 mg of D-luciferine (PerkinElmer, France) in 100 μL of sterile PBS 20 min before, being placed in an atmosphere of 5% vetflurane and 1 g/L of O_2_. During the bioluminescence analysis, the mice were kept anesthetized in an atmosphere containing 2.5% vetflurane and 1 g/L of O_2_. When mice spontaneously died or when they were euthanatized as soon as they exhibited serious signs of illness, their spleens were collected and weighed before being mechanically dissociated and passed through 40 μM nylon sieves (BD Falcon, Franklin Lakes, NJ, USA). After two washes in PBS, the cell content was normalized by adjusting 100 mg of initial spleen weight to 1 mL of PBS volume, and 10 μL was analyzed by microscopy to evaluate the GFP cells content. All animal experiments were performed according to the guidelines of the Institutional Animal Care and Use Committee and the regional ethics committee.

## 3. Results

### 3.1. CMAP Analysis of the shBMI1-K562 Gene Signature

**Figure 1:**
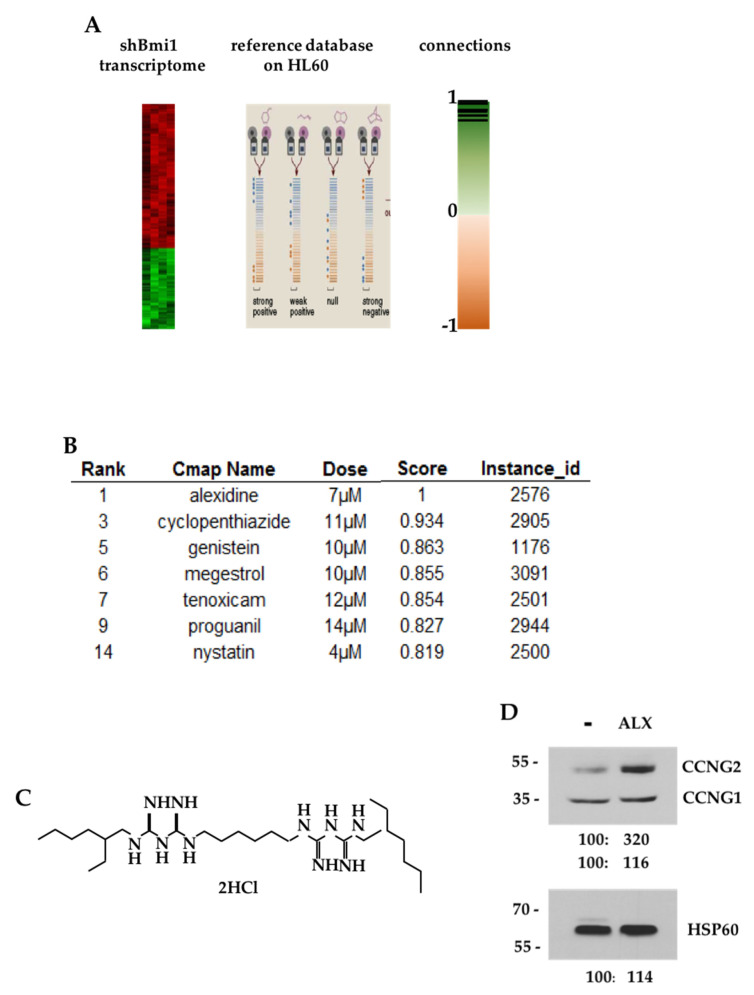
Alexidine was identified by bioinformatic analysis as the best candidate to mimic BMI1 silencing in K562 cells: (**A**) Strategy to identify new candidates mimicking BMI1 silencing in K562 cells among FDA-approved pharmacological molecules referenced in the Connectivity Map database. (**B**) Rankings and scores of ALX and some other molecules after CMAP analysis. (**C**) Structure of ALX. (**D**) K562 cells were incubated 6 h in the presence or absence of 10 μM ALX. Cell lysates were analyzed by immunoblotting for the indicated proteins. Densitometry normalized to control are indicated.

We interrogated the Connectivity Map signature database with a gene expression profile of BMI1-silenced K562 cells and searched for molecules that could mimic BMI1 silencing at the gene expression level, restricting our analysis to signatures obtained by perturbagens acting on HL-60 leukemic cells that were closest to the K562 model (Figure 1A). The highest score was obtained for alexidine (ALX) (Figure 1B)—an alkyl-(bis)-biguanide molecule, the structure of which is shown in Figure 1C. We then verified that ALX could reproduce some functional effects observed after BMI1 extinction and, indeed, we could observe that ALX stimulated the expression of the CCNG2 protein without changing that of the CCNG1 protein in K562 cells (Figure 1D).

### 3.2. Alexidine Synergizes with TKIs to Decrease the Proliferation and Clonogenicity of CML Cells

**Figure 2:**
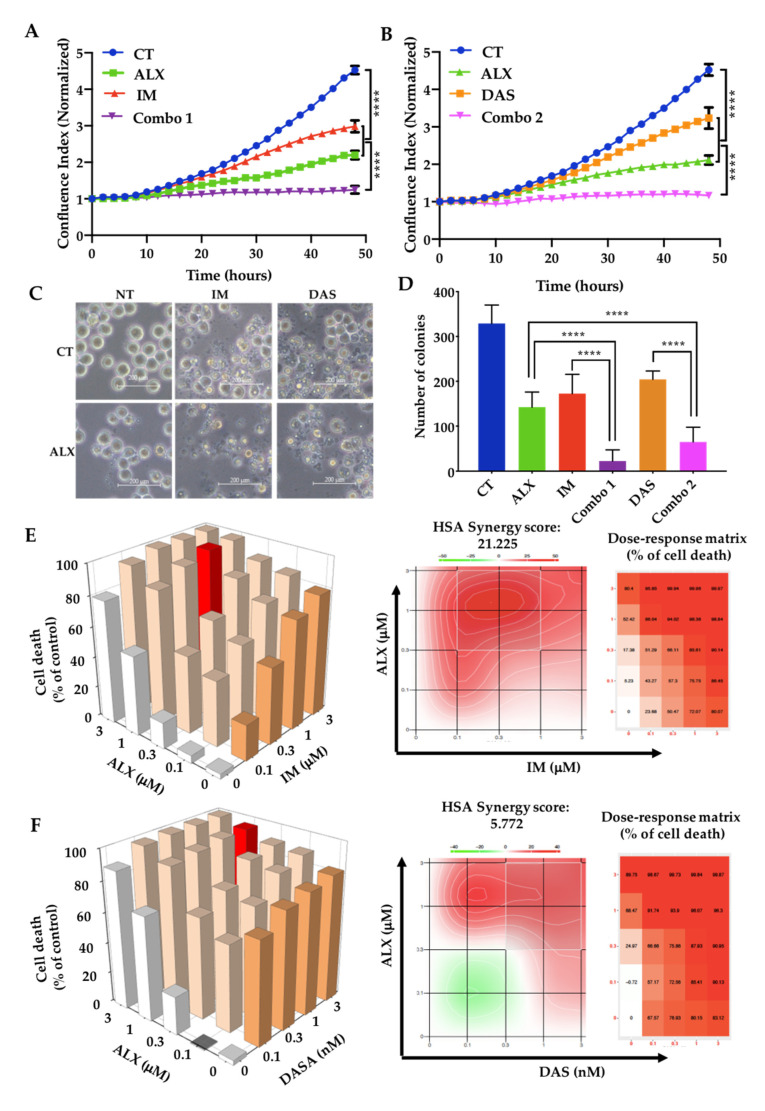
Combinations of alexidine and imatinib or dasatinib inhibited proliferation and induced K562 cell death: (**A**,**B**) Proliferation curves of K562 cells treated with DMSO (CT), 1 µM alexidine (ALX), 1 µM imatinib (IM), 2 nM dasatinib (DAS), and combinations of ALX and IM (Combo 1) or ALX and DAS (Combo 2) for 48 h. Time-lapse analysis of phase-contrast K562 cells using the IncuCyte system. Graphs show quantification of cell numbers from phase-contrast confluence counting. Data are the mean ± SD (n = 3); **** *p* < 0.0001, 2-way ANOVA. A.U. = arbitrary unit. (**C**) Phase-contrast microscopy analysis of K562 cells stimulated for 48 h with the indicated conditions, with DMSO (CT), 1 µM alexidine (ALX), 1 µM imatinib (IM), 2 nM dasatinib (DAS), or combinations of the above. Magnification is 20×, and the scale bar is 200 µM. (**D**) Clonogenic analysis of K562 cells treated for 7 days with vehicle (CT), 1 µM alexidine (ALX), 1 µM imatinib (IM), 2 nM dasatinib (DAS), and combinations of ALX and IM (Combo 1) or ALX and DAS (Combo 2); **** *p* < 0.0001, one-way ANOVA. (**E**,**F**) Cell death 3D graphs (left panel) of K562 cells treated with indicated doses of ALX, IM, and DAS after DAPI staining. Cell death is expressed as the percentage of control. The most valuable ALX/IM and ALX/DAS combinations are represented by the red histograms and were used for all other experiments. The right panels represent synergy density plots displaying the distribution of synergy and the dose–response matrix (right panel) of K562 cells after ALX/IM and ALX/DAS treatments. Combination scores are represented from green (antagonism) to red (strong synergy), following a color gradient. On the dose–response matrix, % of cell death is represented following a color gradient from low to high cell death.

We then observed that ALX combined with IM inhibited K562 cells’ proliferation by 95%, in contrast to 50% and 70% for IM and ALX alone, respectively (Figure 2A). Similar effects were also noted when combining ALX with dasatinib (DAS) (Figure 2B). The combination of ALX and IM was also effective on a second CML line: LAMA-84 (Figure S1). A phase-contrast microscopy analysis of K562 cells showed that ALX, IM, or DAS decreased cell numbers after 48 h of treatment, and more pronounced effects when ALX was added with IM or DAS (Figure 2C). ALX affected the clonogenicity of K562 cells (Figure 2D) in a similar range to IM or DAS, and the combination of ALX with either of these two TKIs completely inhibited colony formation. ALX had similar effects on the LAMA-84 cell line (Figures S2 and S3). We next evaluated the synergistic effects of each combination using the SynergyFinder software. The majority of combinations of ALX with IM or DAS produced several highly synergistic results, with HSA synergy scores of 21.225 for IM/ALX in K562 cells (Figure 2E) and 19.147 in LAMA-84 cells (Figure S4). The DAS/ALX combination in K562 cells gave synergistic results with an HSA synergy score of 5.772 (Figure 2F). Individual synergy score calculation allowed us to identify the best synergy combinations as 1 μM ALX/1 μM IM (Figure 2E, red columns in histograms) and 1 μM ALX/3 nM DAS (Figure 2F, red columns in histograms) in K562 cells. Moreover, we also observed that ALX strongly synergized with the other TKIs—nilotinib (NILO), bosutinib (BOSU), and ponatinib (PONA)—to induce cell death, with HSA scores of about 20 on K562 cells (Figure S5).

### 3.3. Alexidine (ALX) Induces Apoptotic and Autophagic Events

**Figure 3:**
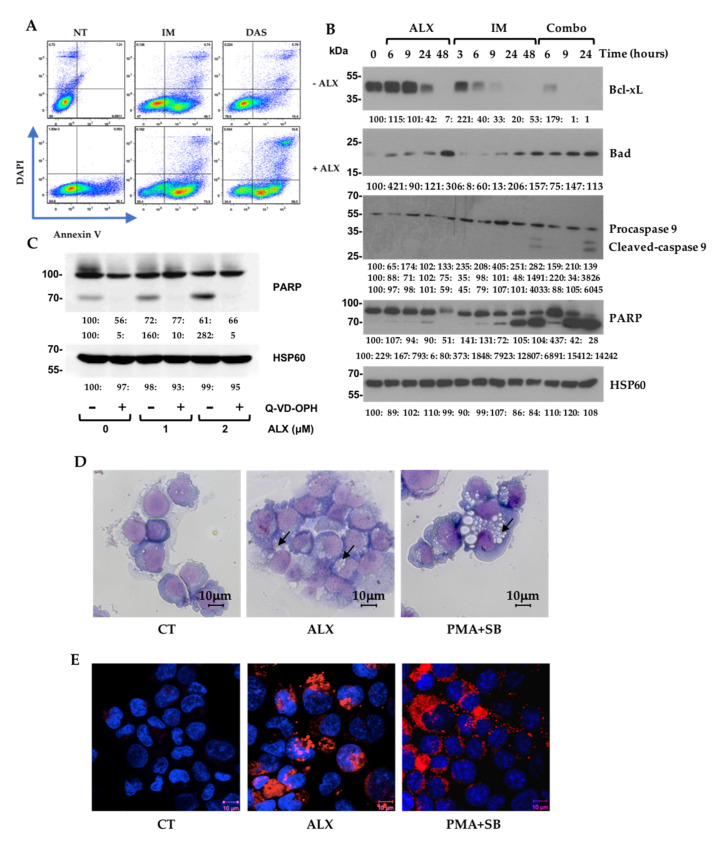
Combinations of ALX and imatinib induced apoptotic and autophagic events in K562 cells: (**A**) K562 cells were stimulated or not with 1 µM alexidine (ALX) alone or in combination with 1 µM imatinib (IM) for 24 h. Cytometry analysis after annexin V/DAPI labeling (**left panel**). Histograms for annexin V+ DAPI+ cells (**top-right panel**) and annexin V+ DAPI− cells (**bottom-right panel**); (**B**) K562 cells were stimulated or not with 1 µM alexidine (ALX), 1 µM imatinib (IM), or their combination (Combo) for the indicated times. Expression of the mentioned proteins was visualized by immunoblotting. (**C**) K562 cells were incubated for 45 min in the presence or absence of 30 μM Q-VD-OPH before being stimulated 24 h with the indicated concentration of alexidine (ALX). Cell lysates were analyzed by immunoblotting for the indicated proteins. (**D**) K562 cells were incubated for 24 h with DMSO (CT), 1 µM alexidine (ALX), or a combination of PMA and SB202190 (PMA + SB). The visualization of vacuolization (arrows) was performed after MGG staining on Cytospin-fixated cells. (**E**) K562 cells stably expressing RFP-LC3 were stimulated for 24 h with DMSO (CT), 1 µM alexidine (ALX), or a combination of PMA and SB202190 (PMA + SB). Cells were analyzed using a confocal microscope. DAPI was used to visualize the nuclei.

To better characterize the cell death pathways underlying the ALX/ITK combination, we first studied the apoptotic pathway by flow cytometry and Western blot on both K562 and LAMA-84 cells. We were able to observe that a 24 h treatment with ALX engaged K562 cells in an apoptotic process, but with a smaller amplitude than IM or DAS. In contrast, the combination of the bis-biguanide with each of the two TKIs caused a strong increase in both the early (AnV+ DAPI−) and late apoptosis (AnV+ DAPI+) (Figure 3A). Similar results were obtained on LAMA-84 cells (Figure S6A). Treatment kinetics with ALX, IM, and their combination allowed us to demonstrate that ALX alone decreased the expression of the anti-apoptotic BCL2 family member Bcl-xL at 24 h, and it strongly increased the expression of the pro-apoptotic Bad at 48 h. Very interestingly, the combination of the two molecules induced the cleavage of PARP as early as 6 h, and up to 24 h (Figure 3B). Using the LAMA-84 cell line, we also observed that ALX alone induced a significant cleavage of caspase-3 from 24 h and up to 48 h, as well as an acceleration of the apoptotic processes at 48 h, with the complete disappearance of the cleaved forms of the caspase-3 and PARP proteins with the combination IM/ALX (Figure S6B). Pretreatment of K562 cells with Q-VD-OPH—a broad-spectrum caspase inhibitor—completely blocked the induction of PARP cleavage by ALX, confirming the involvement of apoptotic pathways in ALX-induced death processes (Figure 3C). To confirm that the bis-biguanide molecule did not simply act on the BCR::ABL1 pathway, we compared its effects with those of IM on the phosphorylation of BCR::ABL1 and STAT5. ALX did not alter the basal phosphorylation of BCR::ABL1 in K562 or LAMA-84 cells, which was totally inhibited by IM (Figure S7). Because we previously showed that the BMI1/CCNG2 axis could regulate an autophagic pathway, we suspected that ALX could also engage cells in an autophagic response after stimulating CCNG2 expression. Indeed, May–Grünwald–Giemsa (MGG) staining of fixed K562 cells revealed that the ALX treatment induced vacuolization (Figure 3D), although to a lower magnitude than a combination of PMA and SB202190 p38 MAP kinase inhibitor that was demonstrated to trigger autophagy in K562 cells [26]. Upon addition of ALX to K562 cells expressing an RFP-LC3, we observed LC3-II foci formation corresponding to the relocalization of RFP-LC3 into autophagosomes (Figure 3E)—a hallmark of autophagy.

### 3.4. Inhibition of Mitochondrial Respiration by Alexidine (ALX) Initiates CML Cell Death

**Figure 4:**
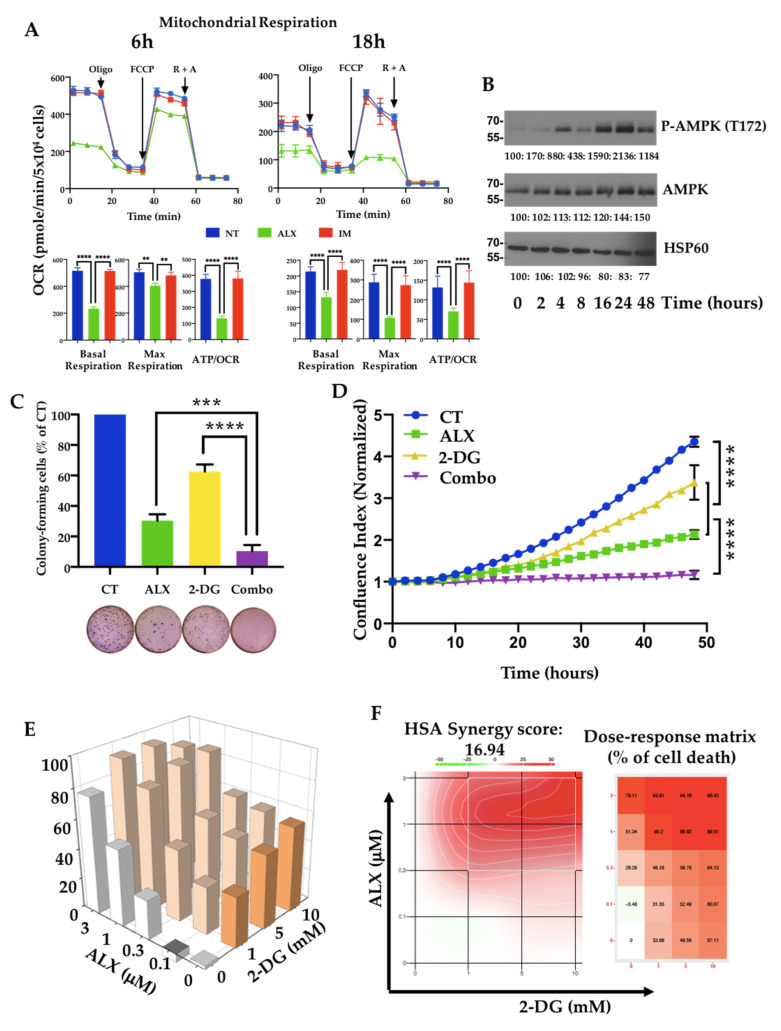
Alexidine inhibited the mitochondrial respiratory chain, leading to leukemic cell death: (**A**) K562 cells were pretreated either with 1 μM alexidine (ALX) or 1 μM imatinib (IM), or left untreated (NT), for 6 h and 18 h, respectively. Oxygen consumption rate (OCR) was evaluated with a Seahorse XF96 Extracellular Flux Analyzer with the Mito Stress test after injection with 1 μM oligomycin (O), 0.5 μM FCCP, and 1 μM concentrations of both rotenone (R) and antimycin A (A). Data are expressed as mean ± SD, representative of 3 independent experiments performed in quadruplicate. Quantification of basal respiration, maximal respiration, and ATP-coupled OCR production (ATP/OCR) is shown in the indicated histograms. Data are expressed as the mean ± SD, representative of 3 independent experiments performed in quadruplicate. ** *p* < 0.01, **** *p* < 0.0001 one-way ANOVA analysis. (**B**) K562 cells were stimulated or not with 1 µM alexidine (ALX) for 24 h. Cell lysates were analyzed by immunoblotting for the indicated proteins. (**C**) Clonogenic analysis of K562 cells treated for 7 days with vehicle (CT), 1 µM alexidine (ALX), 10 mM 2-deoxyglucose (2-DG), or their combination (Combo); *** *p* < 0.001, **** *p* < 0.0001, one-way ANOVA. (**D**) Proliferation curves of K562 cells treated with DMSO (CT), 1 µM alexidine (ALX), 1 µM imatinib (IM), 10 mM 2-deoxyglucose (2-DG), or combination (Combo) for 48 h. Time-lapse analysis of phase-contrast K562 cells using the IncuCyte system. Graphs show the quantification of cell numbers from phase-contrast confluence counting. Data are the mean ± SD (n = 3); **** *p* < 0.0001, 2-way ANOVA. A.U. = arbitrary unit. (**E**) Cell death 3D graphs of K562 cells treated with the indicated doses of alexidine (ALX) and 2-deoxyglucose (2-DG) after DAPI staining. Cell death is expressed as the percentage of control. (**F**) Synergy density plots displaying the distribution of synergy and the dose–response matrix of K562 cells after ALX/2-DG treatments. Combination scores are represented from green (antagonism) to red (strong synergy), following a color gradient. On the dose–response matrix, % of cell death is represented following a color gradient from low to high cell death.

The facts that biguanides such as metformin are known to affect mitochondrial respiration and that ALX targets the mitochondrial PTPMT1 phosphatase, which is known to regulate mitochondrial respiration, led us to analyze the effects of the bis-biguanide ALX on this energetic parameter and on ATP production. Using a Seahorse metabolic analyzer, we observed that basal respiration and ATP production along with OCR were inhibited as early as 6 h into treatment with 1 μM ALX, and maximal respiration was reduced by 80% after 18 h of treatment (Figure 4A). No change in mitochondrial respiration could be detected in the presence of 1 μM IM. The decrease in OCR-linked ATP production in ALX-treated leukemia cells led us to examine the phosphorylation status of the ATP sensor AMPK under these conditions. A time-course exposure of K562 cells to 1 μM ALX showed increasing phosphorylation of AMPK on threonine 172, with a maximum reached after 24 h of treatment, correlating well with the kinetics of ATP production (Figure 4B). We observed that ALX did not affect BMI1 expression but increased CCNG2 expression and totally inhibited the expression of its target, PTPMT1 (Figure S8). IM induced a decrease in BMI1 expression that correlated with a re-expression of CCNG2, but it did not modify PTPMT1 expression. We then associated the inhibition of the mitochondrial respiratory chain by ALX with a blockade of glycolysis by 2-deoxyglucose (2-DG). We first observed that 1 μM ALX together with 10 mM 2-DG drastically decreased both the clonogenic potential (Figure 4C) and the proliferation (Figure 4D) of K562 cells by 90%, while 2-DG alone only inhibited these two parameters by 20%. We performed cross-dosing of ALX and 2-DG for 48 h, quantified cell death by DAPI incorporation followed by flow cytometry (Figure 4E), and calculated the cell death synergies (Figure 4F). We observed that ALX synergized strongly with 2-DG, especially with ALX doses above 0.3 μM.

### 3.5. The Combination of Alexidine (ALX) with a Tyrosine Kinase Inhibitor (TKI) Affects CML Progenitor Cells

**Figure 5:**
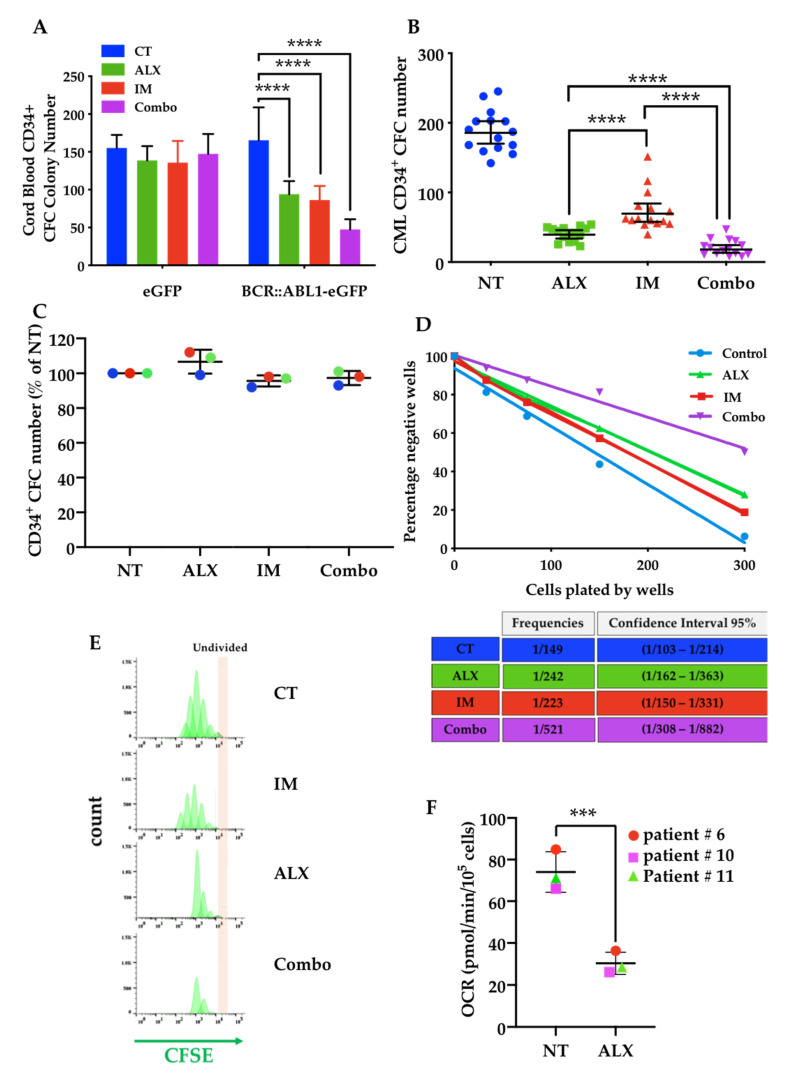
Combination of ALX and Imatinib eliminates CML stem/progenitor cells. (**A**) Evaluation of ALX toxicity. Cord blood CD34+ were transduced with a lentiviral vector expressing *BCR::ABL1* (eGFP- BCR::ABL1) or not (eGFP) and incubated 48 h with DMSO (CT), 1 µM Alexidine (ALX), 1 μM imatinib (IM) or combination of ALX and IM (Combo). The clonogenic capacity of cells was examined after 14 days. Error bars represent SD for 3 biological replicates. **** *p* < 0.0001, 2-way ANOVA analysis. (**B**) Clonogenic capacity of primary CD34^+^ cells from 15 diagnosed CML patients, treated with DMSO (NT), 1 µM Alexidine (ALX), 1 µM imatinib (IM) or combination of ALX and IM (Combo) was examined after 14 days. **** *p* < 0.0001, 2-way ANOVA analysis. (**C**) Clonogenic capacity of primary CD34+ cells from 3 healthy donors (each donor indicated by a color) treated with DMSO (NT), 1 µM Alexidine (ALX), 1 μM imatinib (IM) or combination of ALX and IM (Combo) was examined after 14 days. (**D**) Limited dilution analysis (LDA) of CML LSCs from indicated conditions by LTC-IC assay (Upper panel) and LTC-IC frequencies calculated from LDA (Down panel). (**E**) Primary CD34+ cells from diagnosed CML patient, stained with 5-(and 6-) carboxyfluorescein diacetate succinimidyl diester (CFSE) were cultured in StemSpan without cytokines and treated with DMSO (CT), 1 µM Alexidine alone (ALX), 1 µM imatinib (IM) or combination of ALX and IM (Combo). Cells were harvested at variable time points and labeled with anti-CD34 before evaluation of DAPI+ cells by cytometric analysis. (**F**) Primary CD34+ cells from diagnosed CML from 3 indicated patients were treated with 1 µM Alexidine (ALX) or left untreated (NT) for 24 h. Basal Oxygen Consumption Rate (OCR) was evaluated with Seahorse XF96 Extracellular Flux Analyser, *** *p* < 0.001 one-way ANOVA analysis.

We first evaluated the effect of ALX on the differentiation of primary CD34+ cells derived from human cord blood (Figure S9). The dose response of ALX established the safety of ALX for concentrations below 3 μM, and we selected a dose of 1 μM for the subsequent ex vivo experiments. To evaluate the specificity of ALX’s action on leukemic vs. normal cells, we transduced umbilical cord CD34+ cells with a lentiviral vector encoding the BCR*::ABL1* oncogene. As shown in Figure 5A, ALX selectively inhibited the clonogenicity of BCR*::ABL1* -transduced CD34+ cells similarly to IM, and the combination of the two molecules further increased the inhibitory effect compared to the effect of each of these drugs used alone. We next used bone marrow samples from a cohort of 15 diagnosed CML patients, to assess the impact of ALX alone or in combination with IM on the clonogenic potential of primary CML CD34+ cells. The addition of 1 μM IM led to a mean 60% decrease in the clonogenicity of CML CD34+ cells, whereas 1 μM ALX decreased their clonogenic potential by approximately 70%, with a more homogeneous response compared to IM (Figure 5B). The combination of the two drugs amplified the response, with only 10% of colonies remaining (Figure 5B). Under the same conditions, neither ALX, nor IM, nor their combination affected CD34+ cells from healthy donors, showing their safety for normal cells (Figure 5C). Similar results were obtained by combining ALX with a second- (i.e., nilotinib, dasatinib, or bosutinib) or a third-generation (i.e., ponatinib) TKI (Figure S10). LTC-IC experiments with LDA showed the impact of these treatments on the leukemic progenitor/stem compartment. IM and ALX alone induced a moderate reduction in the frequency of CML stem/progenitor cells, by 1.4- and 1.56-fold, respectively, whereas the combination of the two molecules was very effective in reducing this frequency (3.3-fold) (Figure 5D). Primary CML CD34+ cells were then labeled with the fluorescent cell division tracker CFSE. In the absence of cytokines, the combination of ALX and IM drastically abrogated their proliferation, while the addition of IM or ALX alone only partially decreased the number of dividing CD34+ cells. Cells with the highest CFSE staining corresponding to undivided and most primitive leukemic stem/progenitor cells were eliminated by the ALX/IM combo, while each molecule alone had only modest effects (Figure 5E). Interestingly, we observed that ALX exhibited a strong ability to reduce mitochondrial respiration in primary CD34+ CML patient cells (Figure 5F).

### 3.6. Alexidine Improves the Survival of Leukemic Mice

**Figure 6:**
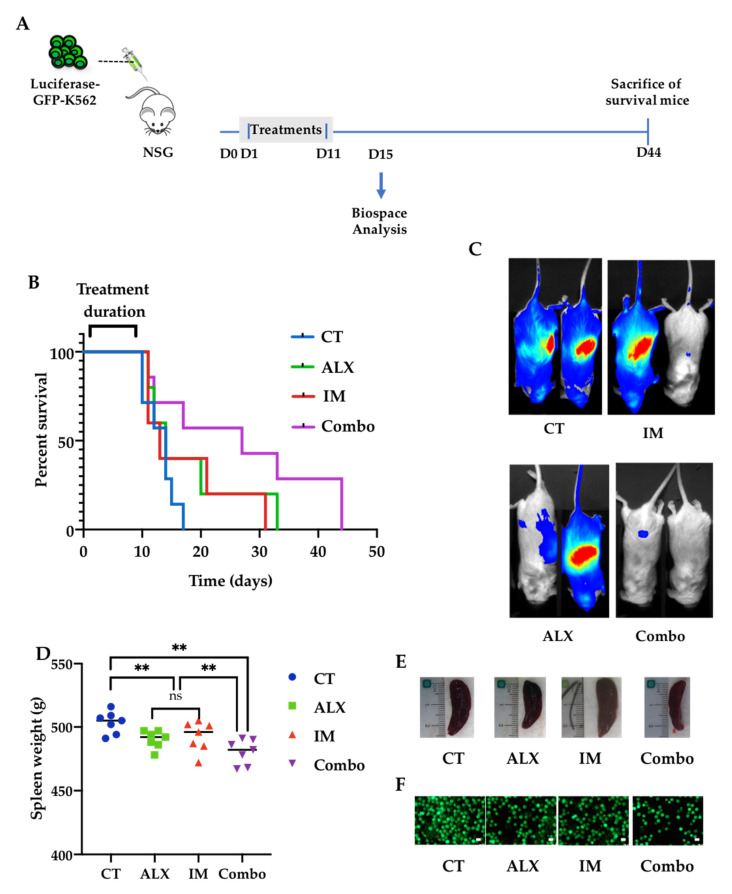
Combination of alexidine and imatinib increased leukemic mice’s survival. (**A**) Schematic design of the in vivo experiment. NSG mice (7 per group) were injected intravenously with GFP-K562-transduced cells. One day later, the mice were treated daily for 10 days with intraperitoneal injection of PBS (CT), 45 mg/kg imatinib (IM), 0.5 mg/kg of alexidine (ALX) or a combination (Combo). The last surviving mice were euthanatized at day 44. (**B**) Survival curves of mice according to the different treatments. (**C**) Two representative images of Biospace analysis of each indicated group of treated mice at day 15. (**D**) Quantification of the spleen weight of all mice in each group (unit = g); ** *p* < 0.01, 2-way ANOVA. (**E**) Photographs representing spleens of mice from each group that died between days 12 and 17. (**F**) GFP cell contents of the spleens presented in (**E**).

In a preliminary experiment, we evaluated the safety of ALX administered by intraperitoneal injection to NSG mice at doses between 0.5 and 4 mg/kg for 12 days. We observed abnormal deaths of the mice between 10 and 12 days with concentrations of ALX up to 1 mg/kg, associated with an increase in the gut mass (Figure S11). We then chose a dose of 0.5 mg/kg ALX to evaluate the anti-leukemic effect of ALX in vivo and compare it to that of IM. NSG mice were intravenously injected with luciferase-GFP-expressing K562 cells, followed by a 10-day period of treatment with PBS, ALX, IM, or ALX/IM before analysis of leukemia progression at day 15 with a Biospace in vivo imager (Figure 6A). The half-life of mice in the control group (11.5 days) was improved by 39% with IM (16 days) and by 30% with ALX (15 days) treatments, and by 82% with the combination of the two molecules (21 days) (Figure 6B). The imaging analysis performed at day 15 showed that leukemic cells were first found in the peripheral blood and then repopulated the spleen, which represents a secondary lymphoid organ devoid of T and B cells in this model. We noted the beginning of a decrease in leukemic invasion both in the peripheral blood and in the spleen in mice treated with IM or ALX alone, and a clear, almost complete disappearance of bioluminescence in mice treated with both molecules (Figure 6C). Upon death or euthanasia, the spleens were weighed, and small statistical differences were observed between the different experimental groups (Figure 6D,E). Nevertheless, the amount of infiltrating GFP-K562 cells in the spleen was decreased to 40% by either ALX or IM, and to 24% by the combination of the two molecules (Figure 6F).

## 4. Discussion

A bioinformatic approach, via the CMAP database, showed that ALX produced a gene expression profile in HL60 leukemic cells that positively correlated with the profile of BMI1-silenced K562 cells. This suggests that ALX could mimic the death-inducing effects triggered by downregulation of BMI1 at the cellular level. Indeed, we found that ALX affected cell proliferation, metabolism, and triggered cell death of CML cell lines and primary cells. ALX also affected CML stem/progenitor cells that represent a clinical issue as they are resistant to Tyrosine Kinase Inhibitors (TKIs) that target the BCR::ABL1 oncogenic driver of CML. Interestingly, ALX showed synergistic cell death effects when combined with TKIs from three generations. BMI1 silencing induced an increase in CCNG2 expression that was also observed with ALX and is likely responsible for the autophagic response observed upon ALX treatment.

ALX belongs to the family of bis-biguanides, similar to biguanides such as metformin and phenformin, which have been used mainly as antidiabetics but were shown in recent years to have anticancer properties as monotherapy or in combination with other chemotherapeutic agents [28]. Metformin, by activating AMPK, has been shown to induce CML cell death [29]. However, its repositioning in anticancer therapy was hampered by the necessity of using high doses that are difficult to reach in vivo. Interest then shifted to phenformin, which could be used at much lower concentrations, but its cytohepatic effects disqualified this drug. During the last few years, several results have emerged highlighting ALX’s efficiency as an apoptosis-inducing anticancer agent in gastric adenocarcinoma [30], head and neck cancer [22], and pancreatic cancer [24]. We found here that it was possible to use ALX ex vivo and in vivo at doses 1000 times lower than those required for metformin, in particular, to reach the leukemia compartment. Beyond an induction of AMPK phosphorylation/activation, we found that ALX acts at two functional levels in the mitochondria of leukemic cells: on the one hand, it allows the dissociation of apoptotic/anti-apoptotic complexes to favor apoptosis, and on the other hand it inhibits mitochondrial respiration. The first molecularly significant consequences of ALX’s action involve the downregulation of anti-apoptotic BCL2 family members—a strategy already envisioned to target LSCs [31] or used in other cancer types [32]. Graber et al. previously described the anti-apoptotic Bcl-xL protein as a direct target of ALX [33]. Nevertheless, in our different model systems used in this study, the IC50 for ALX toxicity was only 2.5 μM, which is four times lower than the doses reported to inhibit Bcl-xL–Bak interactions. Furthermore, Bcl-XL is preferentially required for CML progression [34], while the anti-apoptotic MCL1 is essential for the survival of normal and Ph+ leukemic stem cell populations [35,36], suggesting that ALX must act on additional signaling pathways to target CSLs.

We also found that ALX targets the respiratory electron transport chain (ETC) in the mitochondria—a strategy that is being considered to eliminate LSCs in CML, for instance by using tigecycline, which interferes with translation of some subunits of the ETC [17]. We found that ALX inhibits the expression of its target—the mitochondrial tyrosine phosphatase PTPMT1 (protein tyrosine phosphatase mitochondrial 1) [37]. Furthermore, PTPMT1 expression was demonstrated to be involved in both hematopoietic stem cell (HSC) differentiation [38] and maintenance of long-term HSCs by reprogramming cellular metabolism [39]. Disruption of PTPMT1 expression was shown to severely interfere with mitochondrial functions, leading to inhibition of respiration, reduction in the numbers of ETC complexes, and distortion of mitochondrial morphology [40]. Indeed, ALX targeting PTPMT1 could limit the metabolic reprogramming of LSCs, significantly reducing their capacity to adapt metabolically to the environment. One of the substrates of PTPMT1 is succinate dehydrogenase (SDH)—the second complex of the ETC [41]. Inhibition of PTPMT1 by ALX could increase SDH activity, causing excessive succinate consumption [41]. In addition, PTPMT1 also exerts a regulatory function in the synthesis of cardiolipins that are responsible for mitochondrial membrane integrity [38]. In this context, the inhibition of PTPMT1 by ALX could induce a metabolic crisis that results in LSCs’ death and/or sensitization to the cell death effects of TKIs.

An additional interest in the use of ALX as an anticancer agent is represented by some beneficial effects reported on healthy tissues. ALX has been described to decrease osteolysis induced by mature osteoclasts [42], and to allow the long-term maintenance of HSCs in vivo through the phosphorylation of AMPK on the one hand, and through the inhibition of mitochondrial activity and ROS production on the other hand.

Our results show that ALX could be a promising molecule to target CML LSCs, which represent a reservoir of TKI-resistant cells that can lead to leukemia relapse. Its dual action by interfering with mitochondrial regulation of apoptosis and respiration/energetic metabolism is an original mechanism of action that could more globally affect cancer stem/progenitor cells for wider use in other cancers.

## 5. Conclusions

To the best of our knowledge, this study is the first demonstration that alexidine can target the leukemic stem/progenitor cell compartment in CML. Combination of alexidine with either first-, second-, or third-generation TKIs could provide new treatment options for CML patients.

## Figures and Tables

**Table 1:** Antibodies used in Western blot experiments.

Targets	Antibodies	Ref.	Supplier
AMPK	AMPKα	2532 S	CST
Bad	D24A9	9239	CST
BCR	BCR	3902 S	CST
Bcl-xL	Bcl-xL	2762	CST
BMI1	Bmi1 (F6)	05-637	Merck Millipore (Upstate)
BCR	BCR	3902 S	CST
Caspase-3	Caspase-3	9662 S	CST
Caspase-9	Caspase-9	9502 T	CST
CCNG2	FL-249	sc-851	SCB
CCNG2	N19	sc-7266	SCB
HSP60	HSP60 (K-19)	sc-1722	SCB
HSP90	HSP 90α/β Antibody (F-8)	sc-13119	SCB
P-AMPK	P-AMPK (T172)	2535	SCB
PARP	PARP	9542 S	CST
P-c Abl	P-c Abl (Y245) (73E5)	2868 S	CST
PTPMT1	PTPMT1	sc-390901	SCB
P-STAT5	P-STAT5 (Tyr694) (C11C5)	9359 S	CST
STAT5	STAT5 (D2O6Y)	94205	CST

## Data Availability

Data are contained within the article or the Supplementary Materials.

## Supplementary Materials

The following are available online at https://www.mdpi.com/article/10.3390/cancers15030995/s1, Figure S1: Time-lapse analysis of cells using the IncuCyte system. Figure S2: Combined effects of alexidine and TKIs on LAMA cell death. Figure S3: Clonogenic capacity of LAMA cells under the combination of ALX and TKIs. Figure S4: Alexidine synergizes with imatinib to induce LAMA-84 cell death. Figure S5: Alexidine synergizes with TKIs to induce K562 cell death. Figure S6: Alexidine enhances TKI-induced apoptosis in LAMA-84 cells. Figure S7: Alexidine does not inhibit BCR::ABL1 signaling. Figure S8: CCNG2 and PTPMT1 expressions are inversely regulated by alexidine in K562 cells. Figure S9: Evaluation of ALX’s toxicity to healthy CD34+ cells. Figure S10: Effects of the combination of alexidine and TKIs on CML CD34+ cells’ clonogenicity. Figure S11: Evaluation of ALX’s toxicity to NSG mice.

## Abbreviations

2-DG	2-Deoxyglucose
ALX	Alexidine
AMPK	AMP-activated protein kinase
ANV	Annexin V
ATP	Adenosine triphosphate
Bcl-2	B-cell lymphoma 2
Bcl-xL	B-cell lymphoma extra-large
BCR::ABL1	Breakpoint cluster region-Abelson murine leukemia viral oncogene homolog 1
BMI1	B-cell-specific Moloney murine leukemia virus integration site 1
BOSU	Bosutinib
CCNG2	Cyclin G2
CFC	Colony-forming cell
CFSE	Carboxyfluorescein succinimidyl ester
CSC	Cancer stem cell
CMAP	Connectivity Map
DAPI	Di Aminido Phenyl Indol
DAS	Dasatinib
ETC	Electron transport chain
FDA	Food and Drug Administration
HSC	Hematopoietic stem cell
IM	Imatinib
LIC	Leukemic-initiating cell
LSC	Leukemic stem cell
LTC-IC	Long-term culture of Initiating Cell
Mcl-1	Induced myeloid leukemia cell differentiation protein
MGG	May–Grünwald–Giemsa
NILO	Nilotinib
NSG	Nod Scid Gamma
OCR	Oxygen consumption rate
PONA	Ponatinib
PRC1	Polycomb repressive complex 1
PTPMT1	Protein tyrosine phosphatase mitochondrial 1
RFP	Red fluorescent protein
ROS	Reactive oxygen species
SDH	Succinyl dehydrogenase
SRC	Sarc
TKI	Tyrosine kinase inhibitor
TMRE	Tetramethylrhodamine ethyl ester perchlorate
