# Supplementary material for: Repurposing the Bis-Biguanide Alexidine in Combination with Tyrosine Kinase Inhibitors to Eliminate Leukemic Stem/Progenitor Cells in Chronic Myeloid Leukemia
Source: Cancers (Basel). 2023 Feb 3;15(3):995. doi: 10.3390/cancers15030995 (PMC9913472; doi:10.3390/cancers15030995)
Supplement: Supplementary file 1 [file cancers-15-00995-s001.zip › cancers-2147189-supplementary.pdf]

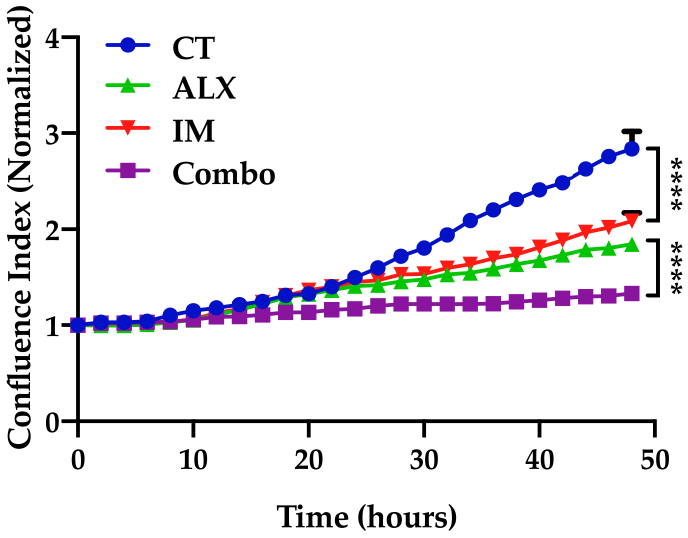

Figure S1. Time-lapse analysis of cell proliferation using the IncuCyte system. Combination of ALX and imatinib on LAMA-84 cell proliferation. LAMA-84 cells were treated for 48h with vehicle (CT), 1µM Imatinib (IM), 1µM Alexidine (ALX) or the ALX/IM combination. Graphs show the quantification of cell number from phase contrast confluence counting. Data are the mean ± SD (n = 3). ****, P < 0.0001, two-way ANOVA analysis. A.U., arbitrary unit.

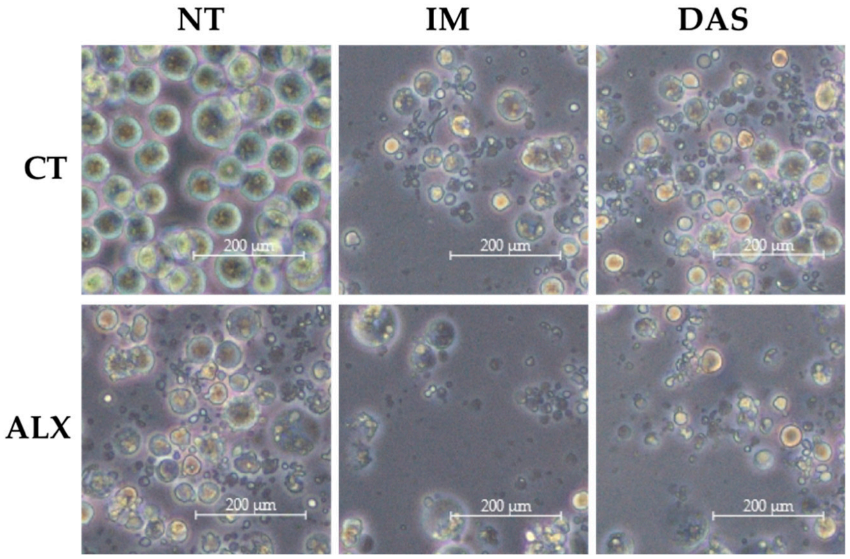

Figure S2. Combined effects of Alexidine and TKIs on LAMA-84 cell death. LAMA-84 cells were stimulated for 48h with vehicle (CT), 1µM Imatinib (IM), 2nM Dasatinib (DAS), 1µM Alexidine (ALX) or combinations and observed by phase-contrast microscopy (Zeiss Axiocam 305 color) 20X. Scale bar is 200µM.

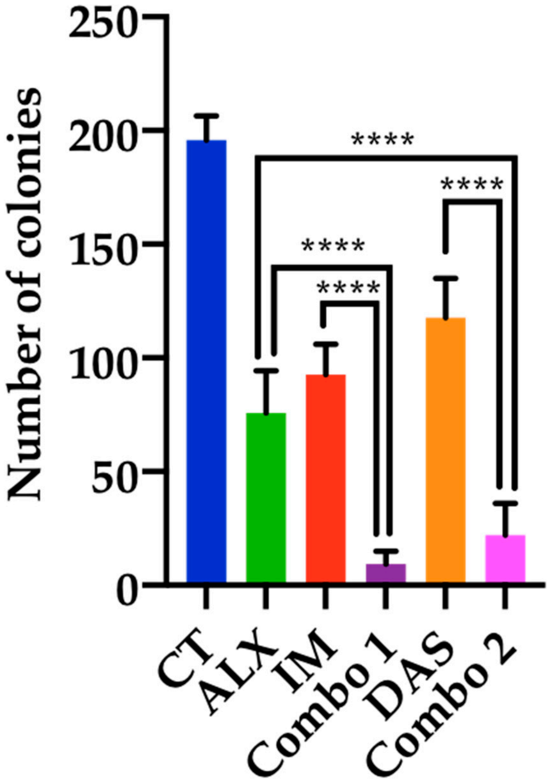

Figure S3. Clonogenic capacity of LAMA-84 cells under combination of ALX and TKIs. LAMA-84 cells were treated for 48h with vehicle (CT), 1µM Imatinib (IM), 1µM Alexidine (ALX), 2nM Dasatinib (Das) or the ALX/IM (Combo 1) or ALX/DAS (Combo 2) combinations. Data are the mean ± SD (n = 3). ****, $P < 0.0001$, two-way ANOVA analysis. A.U., arbitrary unit.

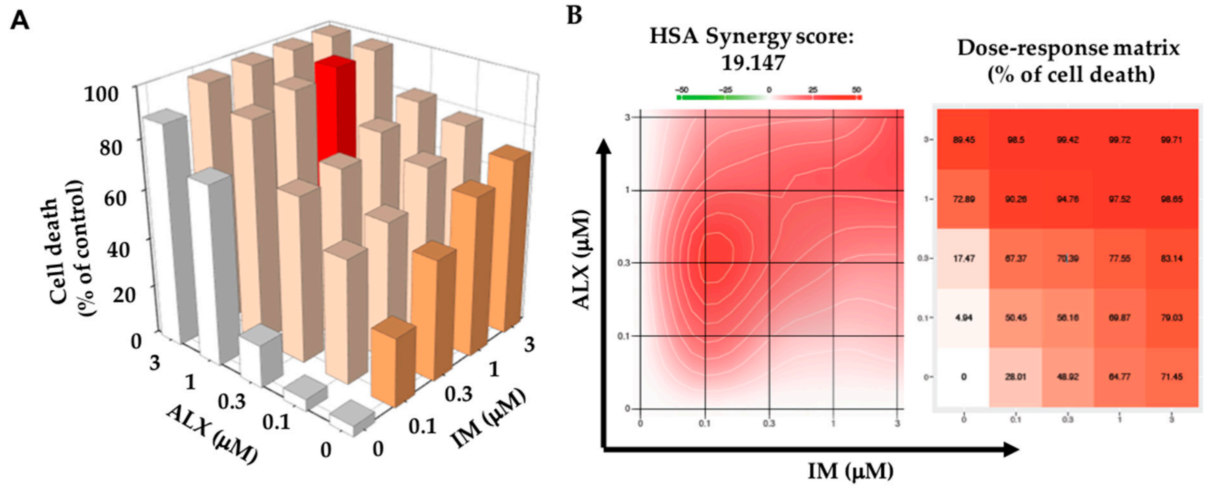

Figure S4. Alexidine synergizes with Imatinib to induce LAMA-84 cell death. 3D graphs visualization of cell death effects on LAMA-84 cells treated with indicated doses of Alexidine (ALX) and Imatinib (IM) measured after DAPI staining. Cell death is expressed as the percentage of control. The best synergistic combinations inducing up to 80% of cell death are represented by the red histograms (left panel). Synergy density plot displaying the distribution of synergy and dose-response matrix (right panel) of LAMA-84 cells after ALX/IM treatments. Combination scores are represented as a color gradient from green (antagonism) to red (strong synergy). On the dose-response matrix, the % of cell death is represented as a color gradient from low to high cell death.

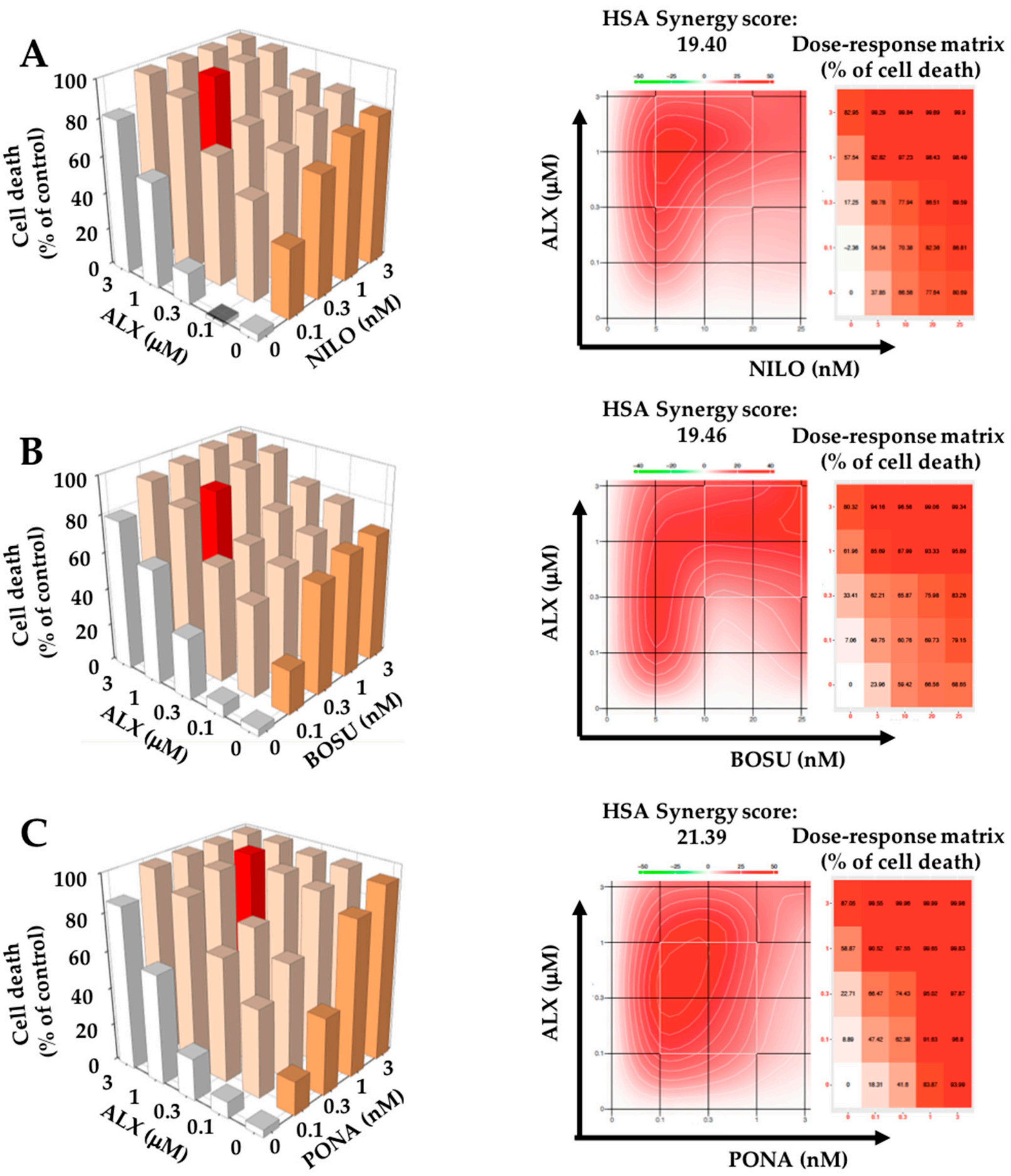

Figure S5. Alexidine synergizes with TKIs to induce K562 cell death. 3D graphs of K562 cells treated with indicated doses of Alexidine (ALX), Nilotinib (NILO) (A), Bosutinib (BOSU) (B) or Ponatinib (PONA) (C) after DAPI staining. Cell death is expressed as percent of control. The best synergy combinations inducing up to 80% of cell death are represented by red histograms. Synergy density plot displaying the distribution of synergy and dose-response matrix (right panel) of K562 cells after ALX/TKIs treatments. Combination scores are represented from green (antagonism) to red (strong synergy) following a color gradient. On the dose-response matrix, the % of cell death is represented following a color gradient from low to high cell death.

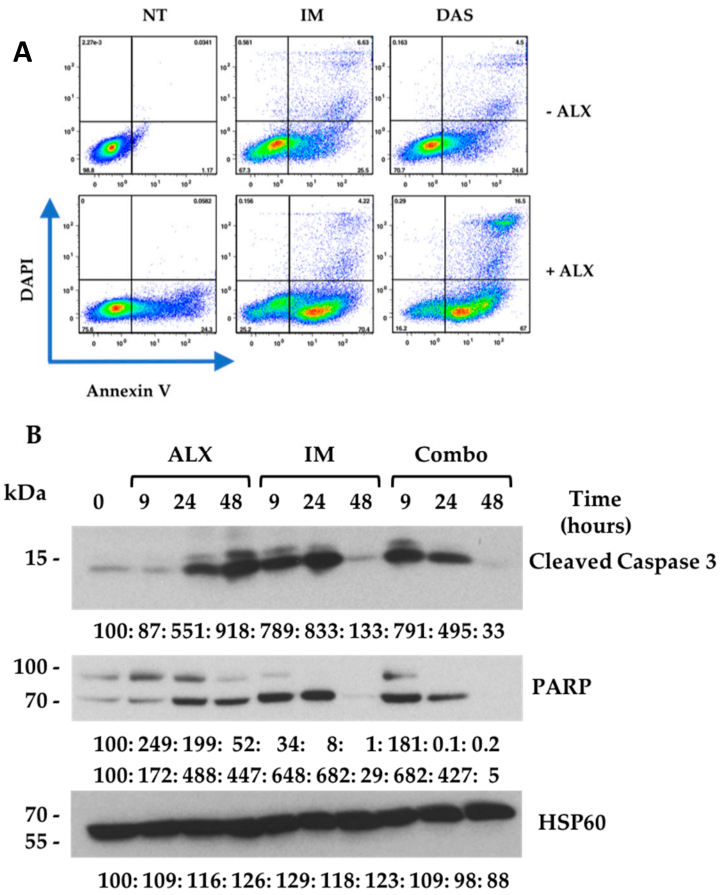

Figure S6. Alexidine enhances TKI-induced apoptosis in LAMA-84 cells. **A.** LAMA-84 cells were stimulated or not (NT), with 1μM Alexidine (ALX) in combination or not with 1μM Imatinib (IM) or 2nM Dasatinib (DAS), for 24hours before cytometry analysis after Annexin V/DAPI labelling. B. Time course of Caspase 3 and PARP cleavage in the presence or not of 1μM Alexidine (ALX), 1μM Imatinib (IM) or the combination of both (Combo). Densitometry normalized to control are indicated.

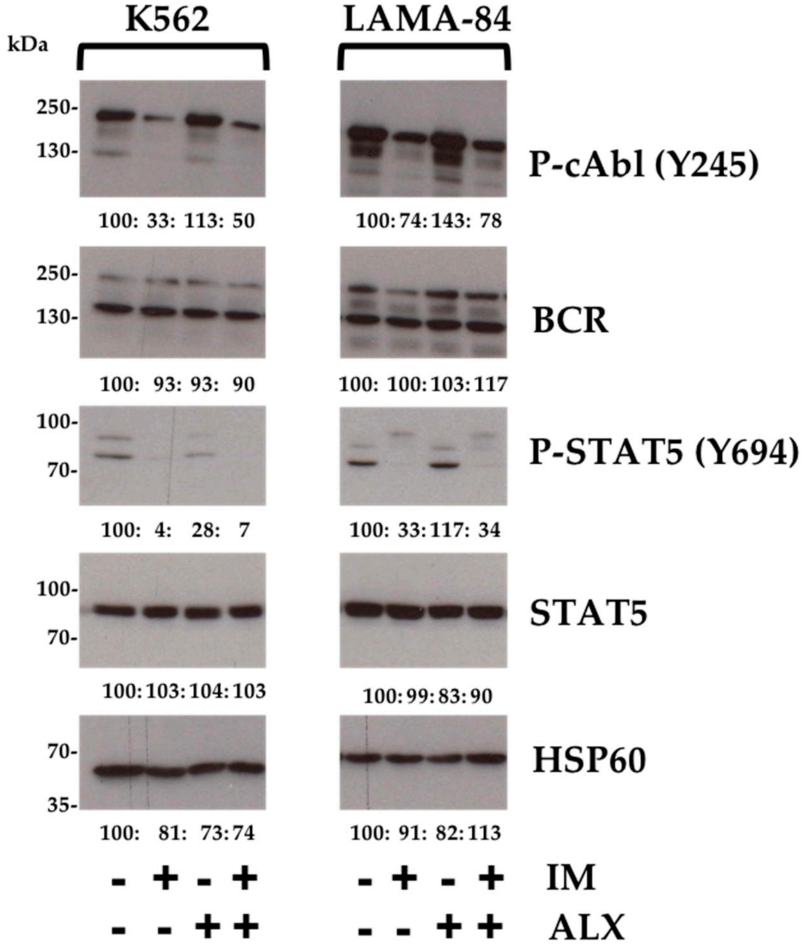

Figure S7. Alexidine do not inhibit BCR::ABL signaling. K562 cells (left) were simulated or not with 1μM Imatinib (IM) or 1μM Alexidine (ALX) for 15 minutes. Cell lysates were analyzed by immunoblotting for indicated proteins. Densitometry normalized to control are indicated.

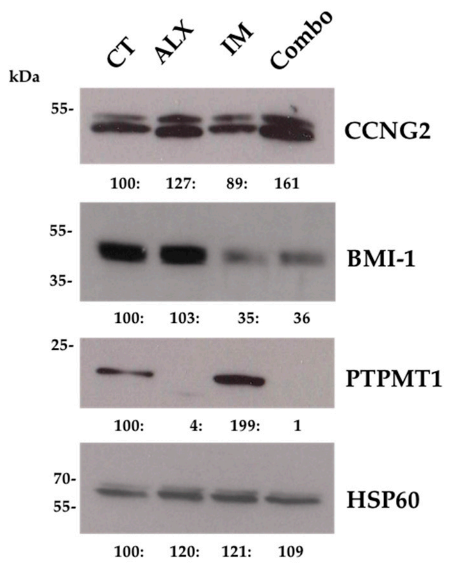

Figure S8. CCNG2 and PTPMT1 expressions are inversely regulated by Alexidine in K562 cells. K562 cells were stimulated or not (CT) with ALX (1µM), IM (1µM) or ALX/IM (Combo). After 24 hours, cell lysates were analyzed for CCNG2, BMI1, PTPMT1 and HSP60 immunoblotting. Densitometry normalized to control are indicated.

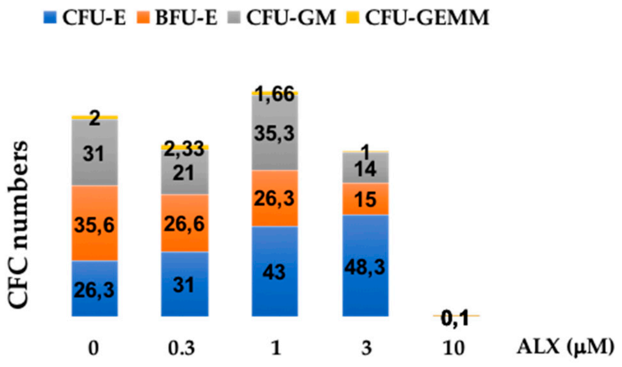

Figure S9. Evaluation of ALX toxicity on healthy CD34+ cells. Human cord blood CD34+ cells were treated or not during 48 hours with the indicated concentrations of Alexidine (ALX) before evaluation of their clonogenic capacity. CFU-GM (Colony Forming Unit-Granulocyte Macrophage), BFU-E (Burst Forming Unit-Erytroid), CFU-GEMM (Colony Forming Unit-Granulocyte Erythrocyte Monocyte Megacaryocyte).

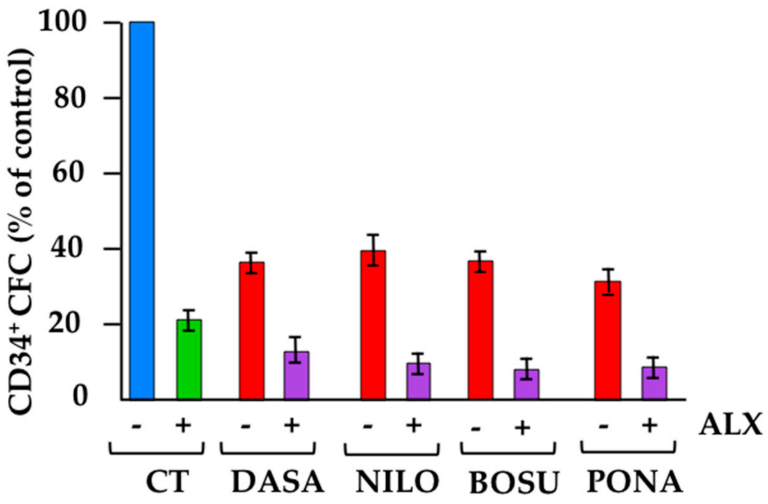

Figure S10. Combination of Alexidine and TKIs on CML CD34+ clonogenicity. Clonogenic capacity of primary CD34+ cells from diagnosed CML patient, treated with DMSO (CT), 2nM Dasatinib (DAS), 2nM Nilotinib (NILO), 10nM Bosutinib (BOSU), 2nM Ponatinib (PONA) combined or not with 1µM Alexidine (ALX) was examined after 14 days.

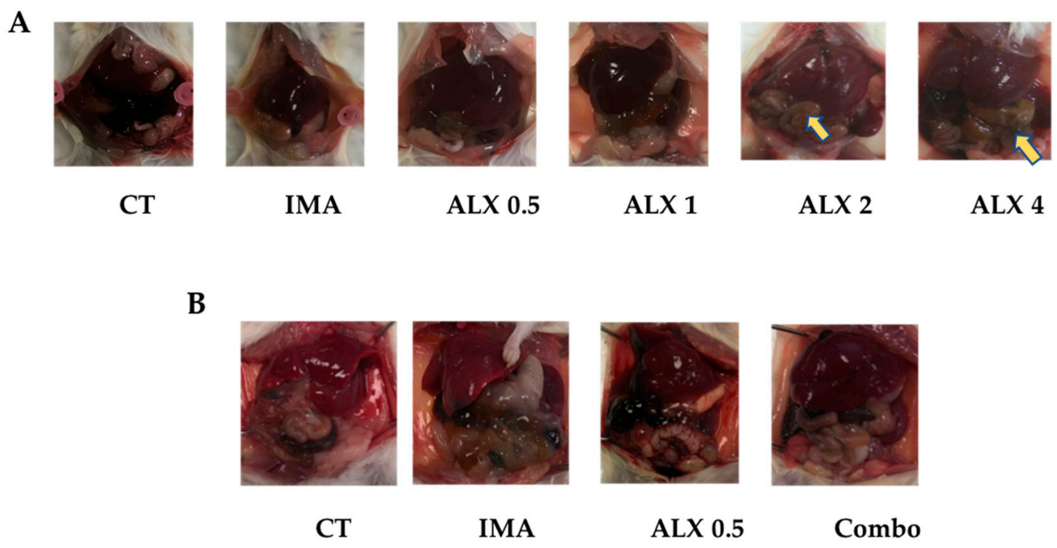

Figure S11. Evaluation of ALX toxicity on NSG mice. **A**.NSG mice were treated daily with intraperitoneal injection of PBS (CT), 45mg/kg imatinib (IM) or increasing concentrations of Alexidine (ALX) (0.5 to 4 mg/kg). The pictures show the abdominal cavity, after spleen ablation, after 10 days of treatment. Arrows show gut inflammation. **B**.NSG mice were treated daily with intraperitoneal injection of PBS (CT), 45mg/kg imatinib (IM), 0.5mg/kg of Alexidine (ALX) or combination (Combo). The pictures show the abdominal cavity, after spleen ablation, after 10 days of treatment.
